# Strategies for reducing the fertilizer application rate in the ridge and furrow rainfall harvesting system in semiarid regions

**DOI:** 10.1038/s41598-017-02731-y

**Published:** 2017-06-01

**Authors:** Yanhao Lian, Xiangping Meng, Zhen Yang, Tianlu Wang, Shahzad Ali, Baoping Yang, Peng Zhang, Qingfang Han, Zhikuan Jia, Xiaolong Ren

**Affiliations:** 1 College of Agronomy, Northwest A&F University, Yangling, Shaanxi 712100 China; 2 Institute of Water Saving Agriculture in Arid Areas of China, Northwest A&F University, Yangling, Shaanxi 712100 China; 3 Key Laboratory of Crop Physi-ecology and Tillage Science in Northwestern Loess Plateau, Ministry of Agriculture, Northwest A&F University, Yangling, Shaanxi 712100 China

## Abstract

The ridge and furrow rainwater harvesting (RFRH) system is a promising water-saving planting technique for dryland farming, but we lack a full understanding of the effects of different fertilizer rates (N:P) on plant nutrient uptake and nutrient use efficiency (NuUE) in foxtail millet using this planting method, as well as the available nutrient residues in the soil. We conducted field studies (Loess Plateau, China) comparing RFRH planting (R) and traditional flat planting (T) at four different fertilizer rates to determine suitable fertilizer application rates for R during 2013–2015. Compared with T, R improved the soil moisture and the utilization of rainwater and fertilizer, thereby enhancing the grain yield, water use efficiency (WUE), grain nutrient uptake, and NUE in a dry year, but with no improvements in a rainy year. The grain yield and WUE exhibited parabolic increasing trends as the fertilizer application rate increased over three years, but no significant increase was found when the fertilizer rate exceeded 189:96 kg N:P ha^−1^ under R, which significantly reduced the NuUE and might waste nutrients. Therefore, we recommend R combined with 189:96 kg N:P ha^−1^ as a promising planting strategy for foxtail millet in semiarid areas.

## Introduction

In the semiarid Loess Plateau region of northwest China, dryland farming is the major agricultural type and crop production depends mainly on limited and erratic precipitation^1^. This region is strongly governed by the monsoon climate, where over 60% of the annual rainfall is received between July and September^2^. Most of the rainfall is low intensity (<5 mm) and it cannot be utilized by crops. In addition, occasional thunderstorms in this region cause great water losses and soil erosion^3^. Strong winds in the spring and high temperature radiation in the summer lead to high rates of soil evaporation^3^. Thus, the development of planting methods is an urgent requirement in order to maximize the water use efficiency (WUE) due to the limited water resources in this area.

The ridge and furrow rainwater harvesting (RFRH) system has been used in semiarid agro-ecosystems to solve the problem of water shortages in China since the early 1990s^4^. This planting method comprises alternate parallel ridges and furrows built along the contours of a field, where the ridge is usually mulched with film for rainwater harvesting and the furrow is used for crop planting without mulching^5, 6^. This method can improve the soil water conditions by collecting water from light rain and retaining surface runoff from heavy rain. Plastic mulching prevents the evaporation of water and prolongs the period of moisture availability to provide sufficient water in the critical growth stages by crops, thereby improving crop production and the WUE^7–9^. Ren *et al*.^10^ found that the RFRH system can improve the soil water content in the 0–100 cm layer by 5–12% under rainfall of 230–440 mm, where the maximum increase obtained in the maize yield was 75% compared with the conventional flat planting system. In a semiarid area, Ding *et al*.^8^ showed that the RFRH system could increase the millet yield by 135.3–108.5% as well as improving the WUE by 6.8–10.3 kg ha^−1 ^mm^−1^ compared with traditional flat planting. Thus, the RFRH system is one of the most efficient methods for maximizing the utilization of rainfall and improving crop productivity in semiarid regions^11^.

In addition to water, fertilizer (nitrogen and phosphorus) is another key factor that determines the crop yield in dryland farming because of its positive effects on improving the leaf area, dry matter content, and WUE^12–14^. However, excessive fertilizer inputs contribute little to further increases in the crop yield, which might lead to excessive water consumption and a waste of fertilizer. Thus, excessive fertilizer application does not facilitate the sustainable utilization of fertilizer resources and it increases the risk of environmental pollution, such as N and P leaching into groundwater, ammonia volatilization into the atmosphere, and N_2_O emissions via microbial denitrification^13, 15–17^. In recent years, strategies have been proposed to reduce fertilizer inputs to an appropriate level that exactly satisfies a crop’s needs^18^. Thus, Liu *et al*.^19^ found that a lower application rate (110 kg N ha^−1^) for maize on ridges and furrows mulched with plastic-film system obtained 82% of the maximum yield, increased the nitrogen use efficiency (NUE), and mitigated the risk of nitrogen losses from the system. Wang *et al*.^14^ also suggested that a moderate fertilizer application rate (210 kg N ha^−1^) in wheat fields could increase the uptake of soil-N and decrease NO_3_-N leaching under supplemental irrigation. However, Zhong *et al*.^17^ considered that it is not appropriate to reduce the nitrogen application rate because a lack of nitrogen can decrease the soil organic carbon contents, thereby decreasing the long-term soil productivity and reducing crop yields. Therefore, the optimal fertilizer application rate should be determined based on analyses of soil residual nutrients to achieve superior crop yields and use resources efficiently.

Furthermore, it has been hypothesized that the soil moisture and fertilizer contents will have synergistic effects on crop growth, and thus if both the water and fertilizer are managed appropriately, their synergistic interaction can increase the crop yield, WUE, and NUE^20–22^. Guo *et al*.^23^ also reported that the optimal N fertilization rates in dry, normal, and wet years were 45, 135, and 180 kg ha^−1^, respectively, in a rain-fed winter wheat cropping system on the Loess Plateau, China. Wang *et al*. found that the RFRH system was beneficial for harmonizing the relationship between soil water and nutrients, and for stimulating nutrient uptake by crops to obtain a high yield^24^. In semi-humid regions, Li *et al*. highly recommended RFRH practices combined with an N rate of 75 kg ha^−1^ as a promising strategy to increase the wheat yield and WUE, which could increase the N fertilizer productivity and N uptake efficiency^9^. Thus, it is necessary to optimize the fertilizer input rate under the RFRH system to obtain further increases in the yield and WUE during dryland farming.

It should be noted that most previous studies of RFRH planting systems did not consider land loss problems. However, sacrificing a portion of the land area to provide water for crop growth is the essence of rainwater harvesting farming, which has been applied in some arid and semiarid regions^25^. In our study, the RFRH system had ridges and furrows with widths of 60 cm, which is suitable for most crops and it facilitates mechanized operations, as well as obtaining better yield increases^8, 26^, although it loses some of the land area. Our research group have performed many studies using this RFRH system since the dryland farming project started in China in 1995, where previous studies focused mainly on water regulation^27^, appropriate precipitation^10^, and cover materials^5^. In the present study, we determined suitable fertilizer application rates for use in this RFRH system. Therefore, we conducted a field fertilizer trial in a dryland agricultural system to elucidate the effects of fertilizer on foxtail millet under the RFRH system, which we compared with the traditional flat planting pattern. The main objectives of this study were as follows: (1) to clarify the effects of the RFRH system on nitrogen and phosphorus uptake by foxtail millet under different fertilizer rates (F0: no fertilizer, F1: N:P at 93:48 kg ha^−1^, F2: N:P at 186:96 kg ha^−1^, and F3: N:P at 279:144 kg ha^−1^); (2) to compare the soil nutrient residue levels (NO_3_-N and available P) under the RFRH system and traditional flat planting with different fertilizer rates; and (3) to identify a highly efficient and sustainable fertilization strategy for use in foxtail millet production with the RFRH system during dryland farming in the semiarid area of China.

## Results

### Soil water content (SWC) profile and soil water storage (SWS)

**Figure 1:**
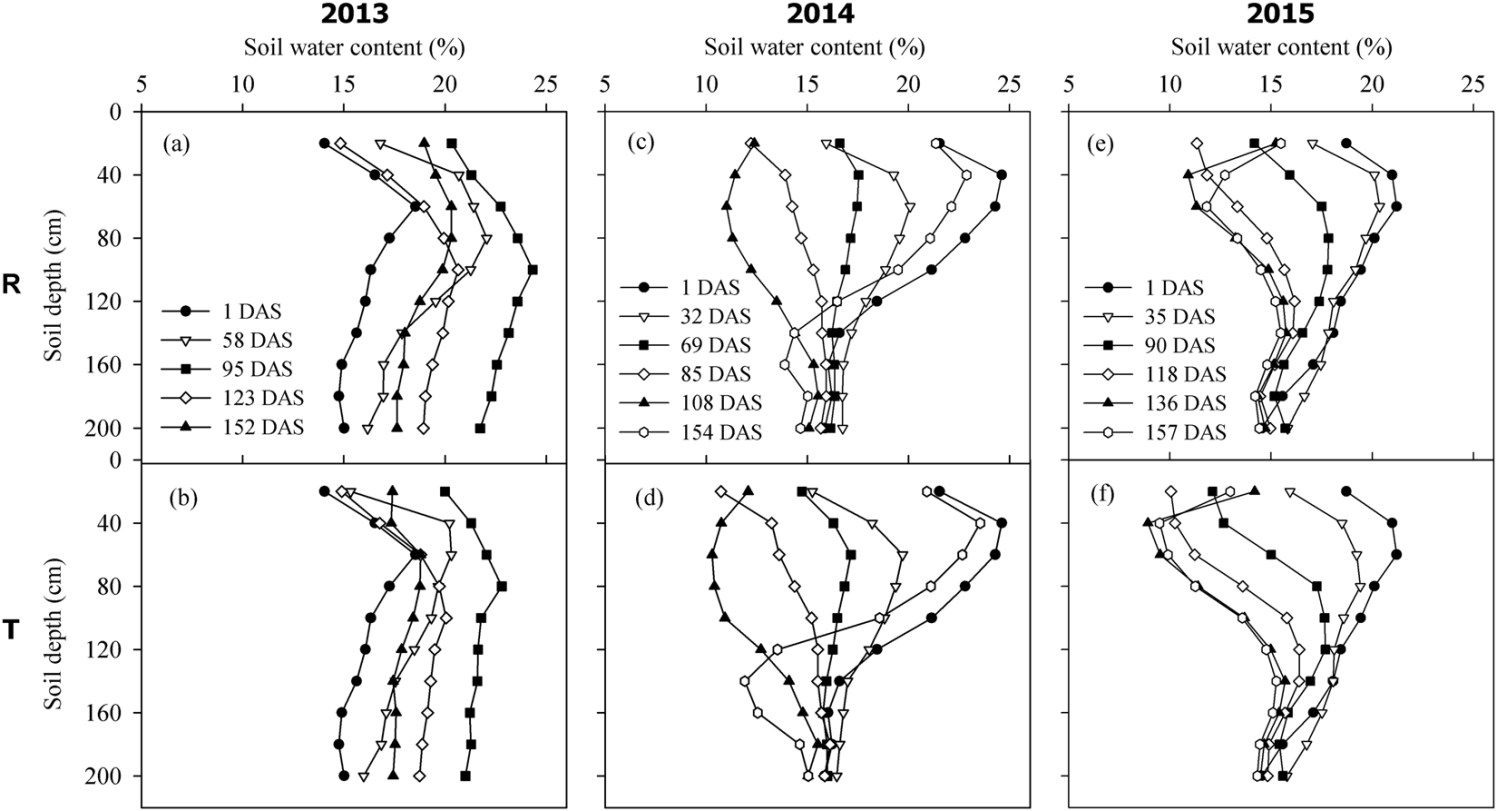
Dynamic variations in the soil water content (%) profile in the 0–200 cm soil layers with days after sowing (DAS) under two planting patterns regardless of the fertilizer rate in the 2013–2015 growing seasons for foxtail millet. Note: R = ridge and furrow rainfall harvesting planting; T = traditional flat planting.

The changes in the SWC profiles in the 0–200 cm soil layers under the two planting patterns are shown in Fig. 1. The differences in the rainfall amounts and distributions during 2013–2015 led to dynamic variations in the SWC profiles. In 2013, the total rainfall was 537 mm and heavy rainfall (189 mm) occurred during 82–89 days after sowing (DAS), which led to deep rainwater infiltration exceeding 200 cm under both planting patterns (Fig. 1a and b), while the SWC remained at values above 15% throughout the whole growing season. Due to insufficient rainfall, the SWC tended to decrease in the 2014 and 2015 growing seasons, and the lowest SWC in the top soil layer was close to the wilting coefficient (8%) at the filling stage, where fluctuations in the soil moisture occurred mainly in the 0–120 cm soil depth. In 2014, 157 mm of rainfall fell during 134–154 DAS (Fig. 1), which led to soil water infiltration to depths of 140 and 120 cm under RFRH planting (R) and traditional flat planting (T) at harvesting (Fig. 1c and d), respectively. In 2015, no deep rainwater infiltration occurred (Fig. 1e and f).

The SWS levels in the 0–120 and 120–200 cm soil depths during the foxtail millet growing season are shown in Table 1. The planting pattern and fertilizer rates mainly affected the SWS in the 0–120 cm soil depth in all three years, whereas the SWS in the 120–200 cm depth was significantly affected by planting pattern at jointing in 2013 and harvesting in 2014, while it was also significantly affected by the fertilizer rate at the filling and harvesting stages in 2014. R improved the average SWS in the 0–120 cm depth by 11–25 mm, 6–12 mm, and 13–30 mm compared with T in the 2013, 2014, and 2015 growing seasons, respectively. At heading (95 DAS) in 2013 and harvesting (154 DAS) in 2014, R significantly increased the average SWS in the 120–200 cm soil depth by 12 mm (*P* < 0.05) and 10 mm (*P* < 0.05), respectively, compared with T. There were only small differences in the SWS among fertilizer rates at the seedling stage, and the SWS in the 0–120 cm soil depth tended to decrease as the fertilizer rate increased, but with R planting, no significant decrease was found when the fertilizer rate exceeded F2 in all three years. In 2014, the SWS in the 120–200 cm soil depth had a similar trend among fertilizer rates at the filling (108 DAS) and harvesting (154 DAS) stages.

**Table 1:** Soil water storage (SWS, mm) at different growth stages according to the fertilizer rate and planting pattern during the foxtail millet growing seasons in 2013–2015.

Year	Planting pattern	Fertilizer rate	SWS at 0–120 cm soil profile (mm)				SWS at 120–200 cm soil profile (mm)			
			Seedling	Jointing	Filling	Harvest	Seedling	Jointing	Filling	Harvest
2013	R	F0	323a	369a	317a	332a	180a	235a	203ab	191a
		F1	317bc	363ab	310ab	321b	176ab	240a	205ab	188ab
		F2	314bc	360ab	300bc	305cd	174ab	236a	205ab	188ab
		F3	315b	355b	293cd	298d	173ab	236a	203a	187b
		**Mean**	**317A**	**362A**	**305A**	**314A**	**176A**	**237A**	**204A**	**188A**
	T	F0	308cd	349bc	306a	311c	180a	223b	199b	186b
		F1	306d	343c	296b	295d	176ab	227b	202ab	185b
		F2	304d	347c	288c	281e	176ab	225b	201ab	185b
		F3	305d	341c	280d	270f	174b	226b	201ab	184b
		**Mean**	**306B**	**345B**	**292A**	**289B**	**177A**	**225B**	**201A**	**185A**
	Analysis of Variance	P	*	*	*	*	ns	*	ns	ns
		F	ns	**	**	**	ns	ns	ns	ns
		P × F	ns	ns	ns	ns	ns	ns	ns	ns
2014	R	F0	300a	243a	201a	337a	179a	169ab	161a	158a
		F1	297ab	229bc	194b	330b	179a	167ab	162a	155ab
		F2	296ab	224bc	187c	325bc	176ab	166ab	158ab	151bc
		F3	296ab	221c	183cd	324bc	178ab	166ab	157b	149cd
		**Mean**	**297A**	**229A**	**191A**	**329A**	**178A**	**167A**	**159A**	**153A**
	T	F0	292bc	230b	189bc	330b	178ab	170a	159ab	147cd
		F1	293bc	219c	179e	325bc	174b	167ab	159ab	144d
		F2	289c	216c	178d	318cd	175ab	165b	157bc	138e
		F3	291c	215c	170e	311d	178ab	166ab	153c	142e
		**Mean**	**291B**	**220A**	**179B**	**321A**	**177A**	**167A**	**157A**	**143B**
	Analysis of Variance	P	*	ns	*	ns	ns	ns	ns	*
		F	ns	**	**	**	ns	*	*	**
		P × F	ns	ns	ns	ns	ns	ns	ns	ns
2015	R	F0	310a	278a	244a	243a	179a	168ab	162ab	156a
		F1	305ab	269b	220c	221b	179a	168ab	159b	156a
		F2	305ab	263b	212d	212c	180a	165b	160b	155a
		F3	301bc	261bc	210d	209c	178a	165b	160b	156a
		**Mean**	**305A**	**268A**	**221A**	**221A**	**179A**	**167A**	**160A**	**156A**
	T	F0	298bc	256c	233b	225b	180a	167ab	165a	157a
		F1	292cd	242de	206d	192d	180a	171a	164ab	157a
		F2	292cd	246d	194e	180e	182a	169ab	162ab	154a
		F3	287d	239e	190e	168f	178a	168ab	162ab	156a
		**Mean**	**292B**	**246B**	**206B**	**191B**	**180A**	**169A**	**163A**	**156A**
	Analysis of Variance	P	*	*	*	*	ns	ns	ns	ns
		F	**	**	**	**	ns	ns	ns	ns
		P × F	ns	ns	ns	ns	ns	ns	ns	ns

Within a column, means followed by the same letter for each year are not significantly different at the 0.05 probability level according to the least significant different test (LSD 0.05); lowercase and uppercase letters indicate comparisons among different treatments and between two planting patterns, respectively. ns denotes no significant different; *significant different at the 0.05 probability level; **significant different at the 0.01 probability level; P is the planting pattern, F is the fertilizer rate. The same symbols and abbreviations are used in the other tables.

### Accumulation of nutrients (nitrogen and phosphorus)

Table 2 shows the amounts of nitrogen (N) and phosphorus (P) accumulated by straw, grain, and the total plant for foxtail millet after harvesting in 2013–2015. The application of fertilizer significantly affected the accumulation of N and P in all three experimental years, where the accumulation of N and P tended to increase with the fertilizer rate, but the relative gains declined. The planting pattern significantly affected the N and P accumulated by straw (SNA and SPA), and the N and P accumulated by grain (GNA and GPA) in 2014 and 2015, but there were no significant differences in the N and P accumulated by the total plant (TNA and TPA) in 2013–2015. Significant interaction effects between the planting pattern and fertilizer rate were also found in 2014 and 2015.

**Table 2:** Effects of planting pattern and fertilizer rate on nitrogen (N) and phosphorus (P) uptake by foxtail millet at harvesting during 2013–2015.

Year	Planting pattern	Fertilizer rate	N uptake (kg ha^−1^)			P uptake (kg ha^−1^)		
			Straw	Grain	Total plant	Straw	Grain	Total plant
2013	R	F0	17.1e	38.3d	55.4e	5.7e	8.6e	14.3e
		F1	36.5d	68.2c	104.7d	8.5d	14.7d	23.2d
		F2	59.2c	86.8b	146.0c	12.6c	22.6c	35.1c
		F3	67.5ab	91.1ab	158.6ab	14.5ab	25.5ab	40.1ab
		**Mean**	**45.1A**	**71.1A**	**116.2A**	**10.3A**	**17.8A**	**28.2A**
	T	F0	16.8e	38.2d	55.0e	6.3e	8.9e	15.2e
		F1	34.7d	69.6c	104.3d	8.7d	14.3d	23.0d
		F2	59.3bc	94.0a	153.2bc	12.9bc	23.6bc	36.9bc
		F3	69.9a	96.1a	166.0a	15.8a	27.5a	43.3a
		**Mean**	**45.2A**	**74.5A**	**119.6A**	**10.9A**	**18.6A**	**29.6A**
	Analysis of Variance	P	ns	*	ns	ns	ns	ns
		F	**	**	**	**	**	**
		P × F	ns	ns	ns	ns	ns	ns
2014	R	F0	32.0f	67.0de	99.0d	1.7f	10.2d	12.0f
		F1	39.8de	89.0c	128.9c	2.4e	13.9c	16.3de
		F2	56.9c	107.5ab	164.4ab	4.0d	16.5ab	20.5bc
		F3	61.3b	111.5a	172.8ab	5.0b	17.3a	22.3a
		**Mean**	**47.5B**	**93.7A**	**141.3A**	**3.3B**	**14.5A**	**17.8A**
	T	F0	35.4ef	63.1e	98.5d	2.0f	10.2d	12.2f
		F1	46.7d	75.1d	121.7c	2.8e	11.0d	13.8ef
		F2	65.8b	95.6bc	161.5b	4.9c	14.2bc	19.1cd
		F3	73.7a	102.7ab	176.4a	5.9a	16.0ac	21.9ab
		**Mean**	**55.4A**	**84.1B**	**139.5A**	**3.9A**	**12.9B**	**16.7A**
	Analysis of Variance	P	*	**	ns	**	*	ns
		F	**	**	**	**	**	**
		P × F	ns	ns	ns	**	ns	ns
2015	R	F0	12.9e	44.2d	57.1e	1.3f	7.9cd	9.2e
		F1	23.4d	78.7b	102.1c	1.9de	12.1b	14.1c
		F2	38.8c	97.3a	136.1b	3.4c	14.7a	18.0b
		F3	48.3b	98.2a	146.6ab	4.4b	15.4a	19.9a
		**Mean**	**30.9B**	**79.6A**	**110.5A**	**2.8B**	**12.5A**	**15.3A**
	T	F0	13.3e	42.1d	55.3e	1.7ef	7.2d	8.9e
		F1	27.3d	60.6c	87.9d	2.6cd	9.3c	11.9d
		F2	45.0b	87.3ab	132.4b	4.2b	13.1b	17.4b
		F3	57.6a	92.6a	150.2a	5.8a	14.0a	19.8a
		**Mean**	**35.8A**	**70.7B**	**106.4A**	**3.6A**	**10.9B**	**14.5A**
	Analysis of Variance	P	**	*	ns	**	*	ns
		F	**	**	**	**	**	**
		P × F	ns	*	*	*	*	*

The TNA ranged between 55.0–166.0 kg ha^−1^, 98.5–176.4 kg ha^−1^, and 55.3–150.2 kg ha^−1^ in 2013, 2014, and 2015, respectively. As the fertilizer rate increased, the SNA increased significantly, with a parabolic growth trend, but for GNA, there was no significant increase when the fertilizer rate exceeded F2 regardless of the planting pattern in all three years. In 2014 and 2015, the TNA under R did not differ significantly between F2 and F3, where R maintained a higher GNA and a lower SNA compared with T under the same fertilizer rate. In addition, compared with T/F1, the GNA increased significantly under R/F1 (*P* < 0.05) by 18.6% and 29.7% in 2014 and 2015, respectively. By contrast, the SNA decreased significantly under R/F2 and R/F3 (*P* < 0.05) by 13.5% and 16.8% in 2014, and by 13.8% and 16.1% in 2015, respectively, compared with T/F2 and T/F3. The TPA ranged between 14.3–43.3 kg ha^−1^, 12.0–22.3 kg ha^−1^, and 8.9–19.9 kg ha^−1^ in 2013, 2014, and 2015, respectively. The TPA exhibited a similar trend to TNA. In 2014 and 2015, the GPA under R/F1 increased by 26.2% and 30.2%, respectively, compared with T/F1, and the SPA decreased significantly under R/F2 and R/F3 by 18.6% and 15.6% in 2014, and by 20.4% and 23.4% in 2015, respectively, compared with T/F2 and T/F3.

### Translocation of pre-anthesis total plant nutrients (nitrogen and phosphorus)

The N and P accumulation rates at anthesis, as well as the translocation amounts and ratios, and contributions to the grain in 2014 and 2015 are presented in Table 3. These results show that the amounts were higher in 2014 than 2015, and at anthesis, the N and P accumulation rates were affected significantly by fertilizer application in both years, where R planting improved the amounts of N and P uptake at the same fertilizer level in 2015. In both experimental years, the N and P translocation amounts and ratios were affected significantly by the fertilizer rate and planting pattern, where R planting significantly improved the translocation amount and ratio compared with T. The translocation amount tended to increase with the fertilizer rate, but there was no significant difference between F2 and F3. By contrast, the translocation ratio tended to decrease and the highest translocation ratio was obtained under R/F1. The N contribution (Nc) of the pre-anthesis total N relative to the grain N ranged from 0.70 to 0.86 g g^−1^ and the P contribution (Pc) ranged from 0.47 to 0.58 g g^−1^ in 2014. In 2015, Nc and Pc ranged from 0.49 to 0.70 g g^−1^ and from 0.21 to 0.39 g g^−1^, respectively (Table 4). In both years, the fertilizer rate significantly affected Nc and Pc, which were only affected significantly by the planting pattern in 2015. Nc and Pc generally decreased as the fertilizer rate increased, and compared with T, the mean Nc and Pc rates under R increased by 5.7% and 4.6% in 2014, respectively, and by 24.0% (*P* < 0.05) and 32.3% (*P* < 0.05) in 2015.

**Table 3:** Nitrogen and phosphorus uptake, translocation (Nt and Pt), and contribution to grain ratio (Nc and Pc) at anthesis according to the fertilizer rate and planting pattern during 2014–2015.

Year	Planting pattern	Fertilizer rate	N uptake (kg ha^−1^)	Nt (kg ha^−1^)	Nc (%)	P uptake (kg ha^−1^)	Pt (kg ha^−1^)	Pc (%)
2014	R	F0	85.7e	53.7de	80.2ab	7.4e	5.6ce	55.2bc
		F1	116.4d	76.5bc	85.9a	10.4d	8.0bc	57.7abc
		F2	137.9bc	81.0ab	75.4b	13.3bc	9.3a	56.2abc
		F3	147.5a	86.2a	77.3ab	14.5a	9.5a	54.7bc
		**Mean**	**121.9A**	**74.3A**	**79.7A**	**11.4A**	**8.1A**	**55.9A**
	T	F0	82.9e	47.5e	75.3b	6.8e	4.8d	47.1c
		F1	110.1d	63.5cd	84.6a	9.8d	7.0c	63.7a
		F2	136.0c	70.2bc	73.4b	12.8c	7.9b	56.0b
		F3	145.8ab	72.1b	70.2b	14.1ab	8.2ab	51.6bc
		**Mean**	**118.7A**	**63.3B**	**75.9A**	**10.9A**	**7.0B**	**54.6A**
	Analysis of Variance	P	ns	*	ns	ns	0.012	ns
		F	**	**	*	**	**	*
		P × F	ns	ns	ns	ns	ns	ns
2015	R	F0	36.3e	23.5d	53.1bcd	4.4e	3.1cd	38.9a
		F1	73.5cd	50.1b	63.7a	6.5c	4.6ab	37.9ab
		F2	107.4b	68.6a	70.5a	8.9a	5.5a	37.5ab
		F3	115.8a	67.4a	68.7a	9.2a	4.8ab	31.1bcd
		**Mean**	**83.3A**	**52.4A**	**64.0A**	**7.3A**	**4.5A**	**36.4A**
	T	F0	31.0e	17.8d	42.3d	3.9e	2.2d	31.0bc
		F1	62.8d	35.6c	58.7ab	5.6d	2.9c	31.6ab
		F2	94.3c	49.3b	56.4bc	7.8b	3.5bc	26.8cd
		F3	103.0b	45.4b	49.1cd	8.7a	2.9cd	20.6d
		**Mean**	**72.8B**	**37.0B**	**51.6B**	**6.5B**	**2.9B**	**27.5B**
	Analysis of Variance	P	**	**	**	*	*	*
		F	**	**	**	**	**	*
		P × F	ns	0.004	ns	ns	ns	ns

**Table 4:** Effects of planting pattern and fertilizer rate on the grain yield (GY), WUE, N and P uptake efficiency (NupE and PupE), N and P use efficiency (NUE and PUE), N and P harvest indices (NHI and PHI), and net economic income (NEI) for foxtail millet during 2013–2015.

Year	Planting pattern	Fertilizer rate	GY (t ha^−1^)	WUE (kg ha^−1 ^mm^−1^)	NupE (kg kg^−1^)	NHI (%)	NUE (kg kg^−1^)	PupE (kg kg^−1^)	PHI (%)	PUE (kg kg^−1^)	NEI (USD ha^−1^)
2013	R	F0	3.80d	9.90c	—	69.1a	68.7a	—	60.1bc	266.3a	2097d
		F1	5.21c	13.31b	1.13a	65.2ab	49.6b	0.25a	63.5a	224.0b	2817c
		F2	6.20b	15.01a	0.78b	59.5bc	42.5c	0.19b	64.2a	176.4c	3287b
		F3	6.37b	15.13a	0.57c	57.4b	40.5c	0.14c	63.7a	160.4d	3285b
		**Mean**	**5.40A**	13.34A	**0.83A**	**62.8A**	**50.3A**	**0.19A**	**62.9A**	**206.8A**	2872B
	T	F0	3.83d	9.15c	—	69.5a	69.9a	—	58.5c	252.2a	2234d
		F1	5.14c	11.99b	1.12a	66.7ab	49.3b	0.25a	62.3ab	223.9b	2896c
		F2	6.49ab	14.46a	0.82b	61.4b	42.0c	0.20b	64.0a	174.6c	3578a
		F3	6.85a	15.01a	0.59c	57.9c	40.7c	0.16c	63.5a	155.9d	3687a
		**Mean**	**5.58A**	12.65A	**0.85A**	**63.9A**	**50.5A**	**0.20A**	**62.1A**	**201.7A**	3099A
	Analysis of Variance	P	ns	ns	ns	ns	ns	ns	ns	ns	*
		F	**	**	**	**	**	**	**	**	**
		P × F	ns	ns	ns	ns	ns	ns	ns	ns	ns
2014	R	F0	4.25d	13.09cd	—	67.7ab	43.9a	—	85.5a	362.2a	2355d
		F1	5.38b	16.06b	1.39a	69.0a	42.5a	0.17a	85.2a	336.1ab	2976b
		F2	5.94a	17.27a	0.88b	65.4bc	37.2b	0.11b	80.5b	298.2c	3201ab
		F3	6.04a	17.42a	0.62c	64.5c	36.4b	0.08c	77.8c	280.9de	3163a
		**Mean**	**5.40A**	**15.96A**	**0.96A**	**66.7A**	**40.0A**	**0.12A**	**82.3A**	**319.3A**	2924A
	T	F0	3.98d	11.62d	—	64.0cd	42.0a	—	84.0a	339.1ab	2321d
		F1	4.77c	13.63c	1.31a	61.6d	37.3b	0.15a	79.5bc	329.1b	2678c
		F2	5.50b	15.12b	0.87b	59.2e	33.5c	0.10b	74.3d	283.0cd	3000b
		F3	5.72a	15.59b	0.63c	58.2e	32.7c	0.08c	73.0d	264.2e	3030ab
		**Mean**	**4.99B**	**13.99B**	**0.94A**	**60.8B**	**36.4B**	**0.11A**	**77.7B**	**303.8B**	2757A
	Analysis of Variance	P	*	*	ns	**	*	ns	*	*	ns
		F	**	**	**	**	**	**	**	**	**
		P × F	ns	ns	ns	ns	*	*	**	ns	*
2015	R	F0	3.01e	7.60d	—	77.4a	62.7a	—	86.0a	388.2a	1701d
		F1	4.83c	11.51b	1.10a	77.1ab	48.8b	0.15a	86.2a	354.4b	2656b
		F2	5.58a	13.04ab	0.73b	71.5bc	41.1cd	0.10b	81.3b	310.4cd	2995a
		F3	5.69a	13.16a	0.53c	67.0de	39.3cd	0.07c	77.7cd	289.8ef	2957a
		**Mean**	**4.78A**	**11.33A**	**0.78A**	**73.2A**	**48.0A**	**0.11A**	**82.8A**	**335.7A**	2577A
	T	F0	2.84e	6.92d	—	76.0ab	58.0a	—	81.4b	360.3b	1655d
		F1	4.18d	9.45c	0.95a	69.1cd	44.5bc	0.13a	77.9c	327.3c	2336c
		F2	5.21b	11.33b	0.71b	66.0d	39.2d	0.09b	75.6cd	299.3de	2833ab
		F3	5.54a	11.81b	0.54c	61.7e	36.5d	0.07c	70.8e	275.6f	2923a
		**Mean**	**4.44B**	**9.88B**	**0.73B**	**68.2B**	**44.5A**	**0.10B**	**76.4B**	**315.6B**	2437A
	Analysis of Variance	P	*	*	**	*	ns	*	**	*	ns
		F	**	**	**	**	**	**	**	**	**
		P × F	*	*	*	ns	Ns	*	*	ns	*

Note: (1 USD = 6.855 Chinese yuan, Bank of China, 2017/2/17).

### Grain yield, resource use efficiency, and net economic income

The grain yield and WUE were significantly affected by fertilizer application in all three years and by the planting pattern in the last two years, i.e., 3.80 to 6.85 t ha^−1^ in 2013, 3.98 to 6.04 t ha^−1^ in 2014, and 2.84 to 5.69 t ha^−1^ under various treatments (Table 4). The WUE exhibited similar trends to the grain yield. In general, higher fertilizer inputs significantly increased the grain yield, but when the fertilizer rate exceeded F2, there was no significant increase under R planting. The interaction between the planting pattern and fertilizer application rate had a consistent effect on the grain yield in 2014 and 2015. However, in 2013, there were no significant differences in the grain yields among the two planting patterns under F0, F1, and F2, where the highest yield was obtained under T/F3, which was significantly higher than that under R/F3. In 2014 and 2015, considerably higher grain yields were obtained under R planting, with significant increases under F1 and F2 compared with T planting. The high fertilizer rate generally had a positive effect on the WUE regardless of the planting pattern, but the effect was not always significant. There were no significant differences in the increases under F2 and F3. In 2014 and 2015, the average WUE under R planting combined with fertilizer (F1, F2, and F3) increased significantly by 14.5% (*P* < 0.05) and 15.7% (*P* < 0.05), respectively, compared with T.

The nitrogen and phosphorus uptake efficiency (NupE and PupE), NUE, phosphorus use efficiency (PUE), and the nitrogen and phosphorus harvest indices (NHI and PHI) varied significantly among different fertilizer rates, where they tended to decrease as the fertilizer input rate increased. However, the differences between the two planting patterns were not always significant in the three years, and there was no significant difference in 2013 (Table 4). In 2014 and 2015, NupE, NUE, and NHI increased on average by 4.5%, 8.2% (*P* < 0.05), and 7.9% (*P* < 0.05), respectively, under R planting compared with T, while PupE, PUE, and PHI increased on average by 8.6% (*P* < 0.05), 5.6%, and 7.0% (*P* < 0.05), respectively.

Fertilization also significantly increased the net economic income (NEI) regardless of the planting pattern over the three year (Table 4). Under R planting, the maximum NEI was achieved using the F2 fertilizer application rate, which increased NEI by 56.8%, 35.9%, and 76.1% compared with no fertilizer in 2013, 2014, and 2015, respectively, and the NEI decreased (*P* > 0.05) as the fertilizer application rate increased further. In addition, the F3 fertilizer application rate obtained the maximum NEI under T planting. In the rainy year of 2013, T planting obtained a higher NEI than R, and the highest NEI was achieved by T/F3. However, in the dry years of 2014 and 2015, R planting increased the NEI compared with T under the same fertilizer application rate, and the maximum NEI was achieved by R/F2.

### Soil NO_3_-N and available phosphorus content

**Figure 2:**
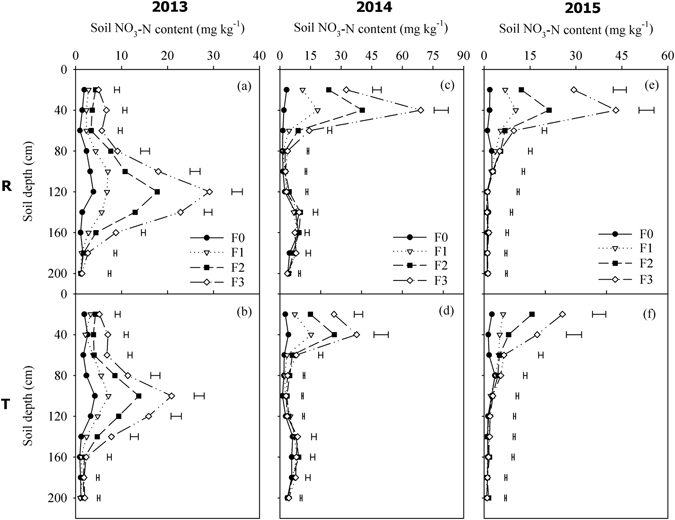
Soil NO_3_-N content at anthesis in the 0–200 cm soil layers according to the fertilizer rate under two planting patterns during 2013–2015. Note: R = ridge and furrow rainfall harvesting planting; T = traditional flat planting; F0, F1, F2, and F3 represent N:P at 0:0, 93:48, 186:96, and 279:144 kg ha^−1^, respectively. Horizontal bars represent the LSD values at the *P* = 0.05 level.

**Figure 3:**
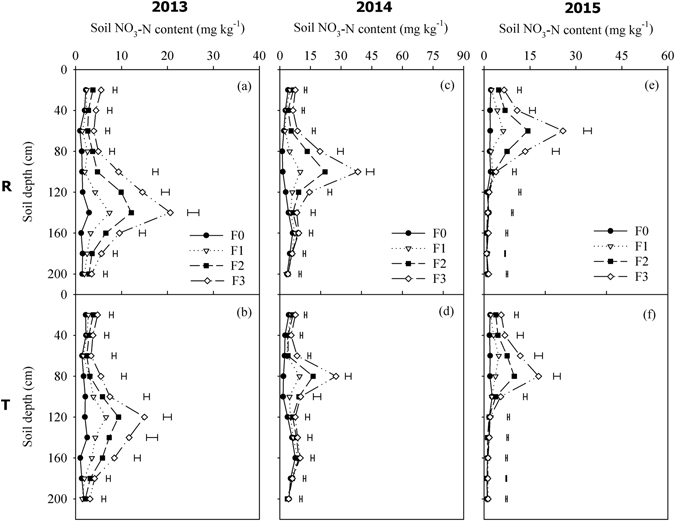
Soil NO_3_-N content at harvesting in the 0–200 cm soil layers according to the four fertilizer rates under two planting patterns during 2013–2015. Note: R = ridge and furrow rainfall harvesting planting; T = traditional flat planting; F0, F1, F2, and F3 represent N:P at 0:0, 93:48, 186:96, and 279:144 kg ha^−1^, respectively. Horizontal bars represent the LSD values at the *P* = 0.05 level.

The dynamics of the soil NO_3_-N content in the 0–200 cm soil layer at anthesis and at harvesting are shown in Figs 2 and 3, respectively. The vertical distribution of NO_3_-N in the soil profile varied among different years, where it was related to the depth of soil water infiltration (Fig. 1). In all three experimental years, high fertilizer application rates significantly increased the accumulation of NO_3_-N in the soil layers, where R planting considerably increased the soil NO_3_-N content and leaching depth compared with T, especially under F2 and F3. At anthesis, the NO_3_-N was distributed mainly in the 0–40 cm soil layer in 2014 and 2015 (Fig. 2c–f), but a large amount of NO_3_-N leached into the deep soil layer below 80 cm in 2013 (Fig. 2a and b). At harvest time, the NO_3_-N leached further into the deeper soil layer, where the peak NO_3_-N accumulation rates under R planting occurred in the 140 cm, 100 cm, and 60 cm soil layers, and the highest NO_3_-N contents reached 20.6, 38.2, and 25.8 mg kg^−1^ under F3 in 2013, 2014, and 2015, respectively (Fig. 3a,c and e). With T planting, the highest NO_3_-N contents occurred in the 120 cm, 80 cm, and 80 cm soil layers, where they reached 15.0, 27.5, and 17.8 mg kg^−1^ in 2013, 2014, and 2015, respectively (Fig. 3b,d and f).

**Figure 4:**
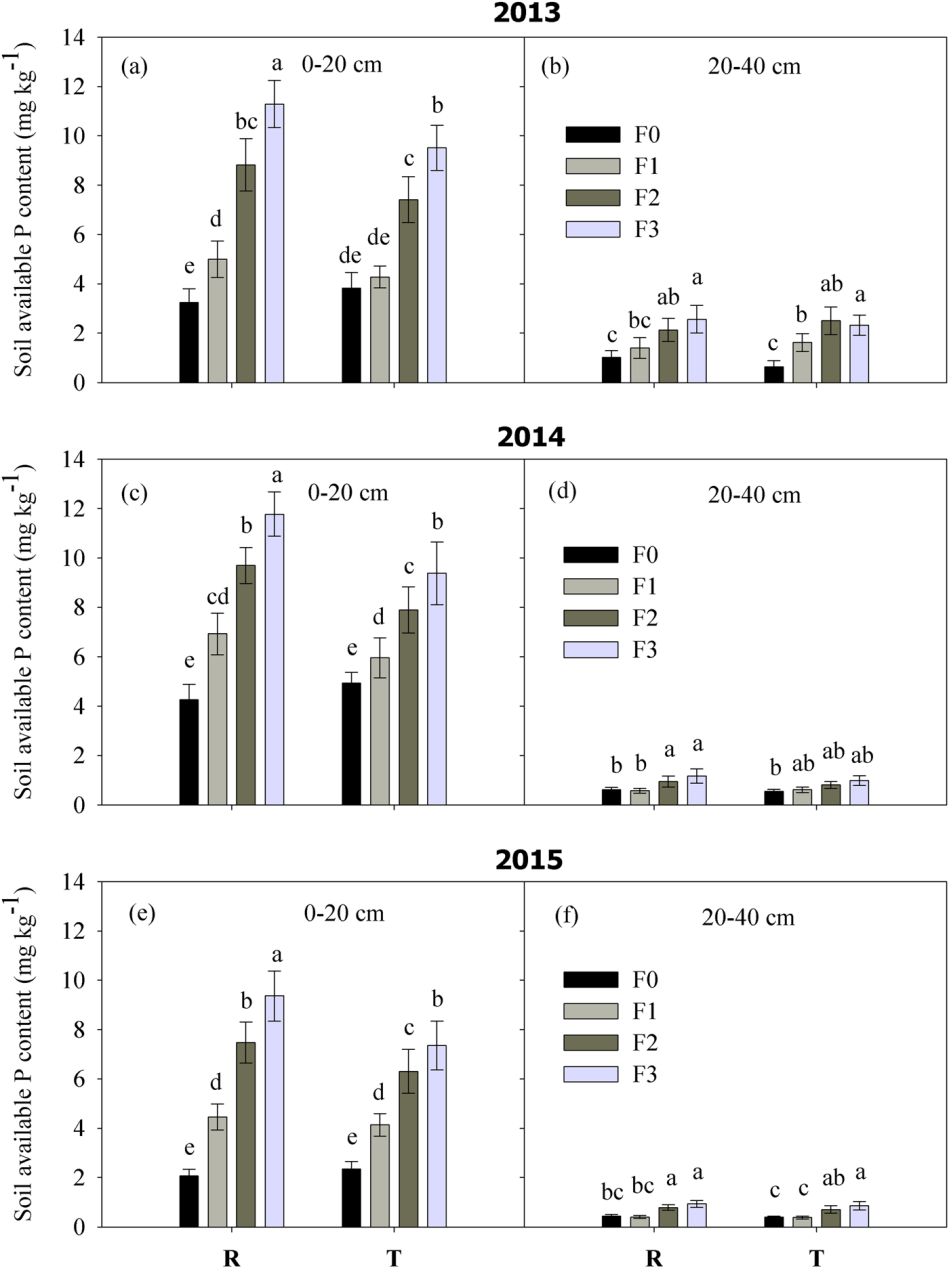
Soil available P contents in the 0–20 cm and 20–40 cm soil layers after harvest according to the fertilizer rate and planting pattern during 2013–2015. Note: R = ridge and furrow rainfall harvesting planting; T = traditional flat planting; F0, F1, F2, and F3 represent N:P at 0:0, 93:48, 186:96, and 279:144 kg ha^−1^, respectively. Different letters indicate significant differences at the *P* = 0.05 level.

The available soil P was distributed mainly in the 0–20 cm soil layer, as shown in Fig. 4, and the available P content in the 20–40 cm soil layer was less than 2.0 mg kg^−1^, except in 2013. The variation among the different treatments was similar in the three years. The available P content in the 0–20 cm soil depth tended to increase as the fertilizer application rate increased. Compared with T planting, R significantly increased the available P content in the topsoil when the fertilizer application rate exceeded F1. High available phosphorus residue levels were found under R/F3 in all three years, where the available P contents were 11.3, 11.8, and 9.36 mg kg^−1^ in 2013, 2014, and 2015, respectively.

## Discussion

**Figure 5:**
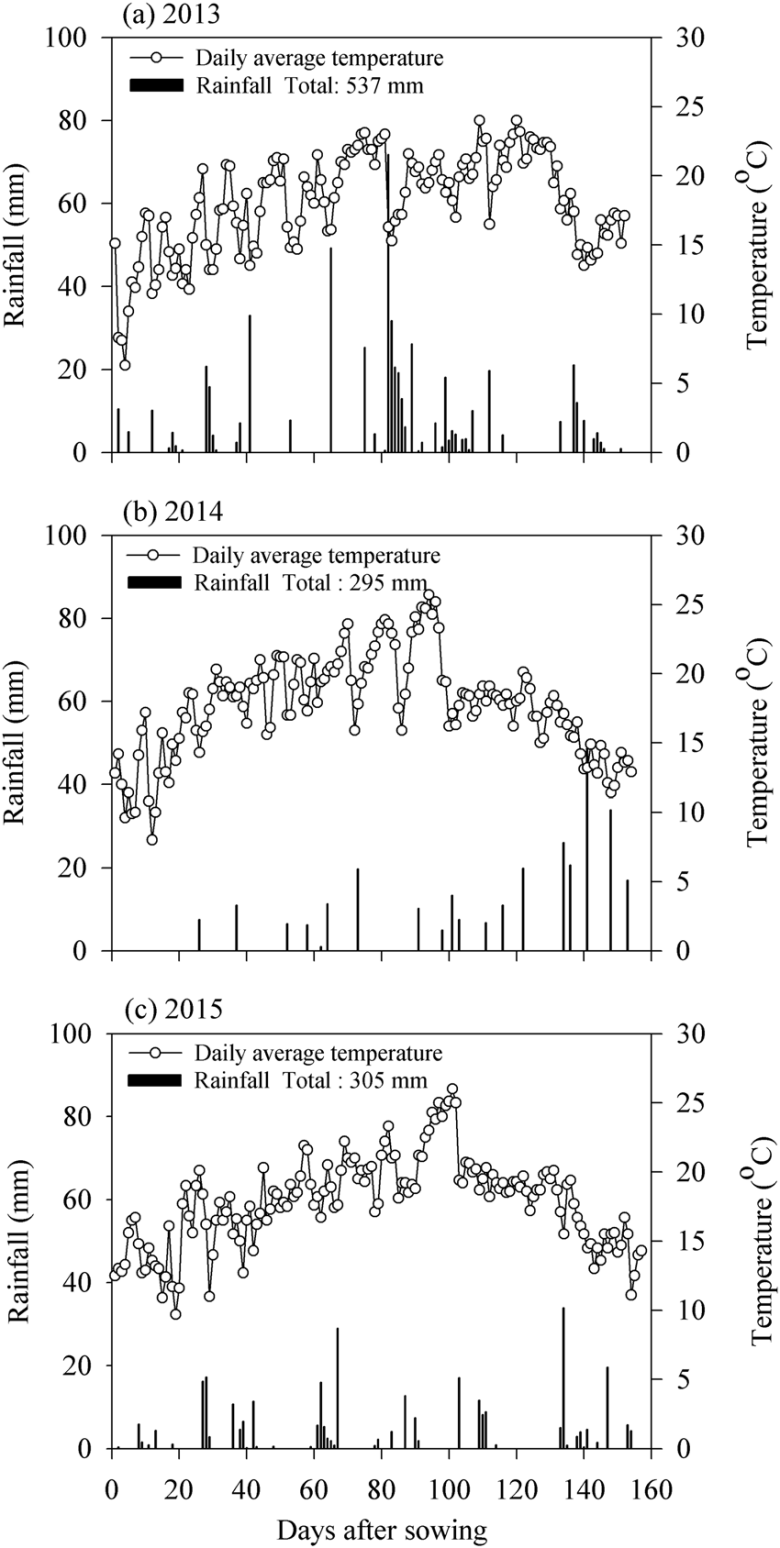
Daily average temperatures and rainfall during the foxtail millet growing seasons in the experimental fields at Pengyang Experimental Station, Ningxia Province, China in 2013–2015.

Rainfall is the key to successful agricultural production in dryland farming. The rainwater harvesting system is an effective method for utilizing rainfall and improving the soil moisture content in semiarid regions^1, 10^. Our results indicated that R planting mainly improved the SWS in the top 0–120 cm depth compared with T, as also reported by Wen *et al*.^28^. In addition, we detected significantly higher SWS levels in the 120–200 cm soil layer under R planting at the jointing stage in 2013 and at the harvesting stage in 2014 when continual and strong rainfall occurred (Fig. 5). Many studies have shown that the application of fertilizer can promote the absorption of soil water^29^. Similar results were obtained in our study, as shown in Table 1, which suggests that fertilizer application favoured biomass growth and increased the transpiration rate, thereby reducing the available soil water^9, 19, 30^. A higher fertilizer application rate leads to higher crop water consumption and lower residual SWS, and our results also showed that were no significant reductions in the SWS when the fertilizer level exceeded F2 under the R planting pattern over the three years, thereby indicating that F2 was sufficient for the growth of foxtail millet. We also found that fertilization had greater effects on the SWS in 2013 and 2015 compared with 2014, which was related to the soil’s basic fertility in each year.

The application of fertilizer can effectively increase the accumulation of plant nutrients, thereby indicating pronounced nutrient limitations for foxtail millet in some areas^9, 31^. According to the present study, F2 (186 kg N and 94 kg P ha^−1^) could meet the nutrient requirements for crop growth regardless of the planting pattern, and only slight increases in the N and P uptake rates by grain were found with higher fertilizer input rates. In dry years (2014 and 2015), our results indicated that pre-anthesis N translocation into the vegetative organs was the main source of GNA, accounting for more than 50%, and similar results were reported by Qiu *et al*.^32^ for winter wheat. However, the source of GPA was mainly attributable to P accumulation after anthesis. The results also showed that the amounts of translocated N and P did not increase significantly or decline with fertilizer application rates above F2. Moreover, the N and P translocation ratio and contribution decreased, which suggests that the excessive application of fertilizer does not facilitate nutrient translocation from the vegetative organs to grain, thereby reducing the NHI, which is consistent with previous findings^33–35^. In our study, we found no significant effects of the planting pattern on TNA and TPA. However, in dry years (2014 and 2015), R planting significantly improved the GNA and GPA compared with T planting, which was achieved in two main ways. First, the improved accumulation of plant nutrients at anthesis facilitated vegetative growth. Under the R planting pattern, the fertilizer and rainwater were concentrated in the planting furrow, which enhanced the absorption of nutrients (N and P) by plants. Second, R planting increased the amount and ratio of nutrients translocated from the vegetative organs to the grain, which is not consistent with the findings reported by Qiu *et al*.^32^. At the experimental site, low temperature limited nutrient translocation in the late growth stage for foxtail millet. R planting can accelerate the duration of plant growth due to the heat-retaining effect of plastic mulching^1, 9^. Compared with R, it was more difficult for foxtail millet to reach complete physiological maturity under T planting, thereby leading to relatively higher nutrient residues in the straw at harvest.

We found that fertilization had a major effect on the production of foxtail millet regardless of the year, where the highest grain yield of 6.85 t ha^−1^ was achieved under T planting combined with the highest fertilizer application rate in 2013, which might be the yield ceiling for foxtail millet under current conditions. In dry years, the maximum yield was achieved under R/F3, but no significant difference in yield was found between R/F2 and R/F3, which indicates that 186 kg N 94 kg P ha^−1^ is sufficient for foxtail millet under RFRH planting in this area. It has been reported that an adequate supply of fertilizer is critical for fully exploiting the benefits of summer rainfall, and the WUE also improved dramatically compared with that without fertilizer, which correlated with the grain yield^36^. Under R planting, there is a trade-off between the ridge width reducing the annual grain yield, and rainwater harvesting by ridges increasing the soil moisture content, grain yield, and WUE^37^. Thus, when the rainfall is lower than a threshold amount, R planting can improve the grain yield, but when the rainfall exceeds this threshold, R planting is unnecessary and the ridges are not useful because soil water is not a key factor that constrains crop growth^1^. Li *et al*.^8^ indicated that it is unnecessary to employ R planting for foxtail millet production in regions with more than 400 mm rainfall. Our results also showed that the use of R planting in dry years (2014 and 2015) significantly improved the grain yield, WUE, and nutrient use efficiency for foxtail millet compared with T planting, which is consistent with previous findings^5, 8, 38, 39^. However, there was no significant improvement in the grain yield under R planting in the rainy year (2013) and there was even a remarkable reduction in the yield with an adequate fertilizer application level. In dry years (2014 and 2015), we found that significant interactions between the planting pattern and fertilizer rate affected the grain yield and WUE for foxtail millet in the present study. Our results also showed that R planting could obtain higher increases in the yield and WUE compared with T planting under a relatively low fertilizer level, which could have two explanations. First, R planting may be more effective under low fertilizer rates, which could enhance the uptake and utilization of nutrients (Table 2). Second, compared with T planting, R planting may be considered a quantitative measure for improving the moisture content, where the maximum synergistic effect can be obtained when combined with an appropriate fertilizer rate.

It has been widely reported that precipitation/irrigation and fertilization influence NO_3_-N leaching in farmland ecosystems^40, 41^. In the present study, the application of N fertilizer at higher than 93 kg ha^−1^ greatly increased the accumulation of NO_3_-N in the soil, which was gradually leached by precipitation into deeper soil layers, especially in the rainy year of 2013 (Figs 2 and 3). It is difficult for summer crops to use NO_3_-N in deep layers because their main root systems are distributed above the 60 cm soil depth, thereby indicating a risk of NO_3_-N leaching^42^. Under R planting, the fertilizer was concentrated in the planting furrow, which occupied only half of the total area, so the excess fertilizer would lead to more NO_3_-N and available P accumulating in the soil compared with T planting. Furthermore, it should be noted that the leaching depth was deeper under R planting, which was caused mainly by the deeper soil infiltration compared with T planting^43^. Therefore, it is necessary for R planting to be combined with an appropriate fertilizer rate in order to reduce the risk of N losses. Feng *et al*.^44^ found that fertilization under the ridge could reduce the risk of NO_3_-N leaching as well as improving the fertilizer use efficiency with R planting, which is highly recommended. Due to the low diffusion coefficient and poor mobility of phosphorus in soil^45^, it is always difficult for crops to absorb phosphorus from the soil. We found that the available P in the soil was concentrated mainly in the top 0–20 cm layer of the topsoil (Fig. 4), where the P content was related to the fertilizer application rate, basic soil fertility, and precipitation. It should be noted that the plant P accumulation rate in 2013 was considerably higher than that in both 2014 and 2015, which is probably attributable to the abundant rainfall in 2013, where the higher soil moisture increased the available soil P content and facilitated the uptake of P by crops. In the present study, using F1, the soil residual available P content at harvest ranged from 4.5 to 6.9 mg kg^−1^ under R planting, which is a moderate level for a calcareous soil in China^45^. The excessive application of fertilizer (nitrogen and phosphorus) would lead to high NO_3_-N and available P residual levels, which might not be environmentally friendly and sustainable in the long term, with possible negative effects on farmland ecosystems.

In recent years, achieving a high yield has not been the only target of agricultural production because shortages of water and fertilizer resources have made it more important to prevent resource wastage and to maintain the sustainable development of agricultural ecosystems^46^. In the present study, the fertilizer was spread over the whole planting furrow, which only occupied half the area under the RFRH system, and thus the fertilizer application rate per unit area was doubled, i.e., the fertilizer rate in the planting furrow in the R/F3 plot was 558 kg N 288 kg P ha^−1^, and this is an excessive level in semiarid regions. In dry years, we found that the RFRH system obtained a better yield than T under the same fertilizer application rate, and when the fertilizer application rate was reduced to F2, there were no significant reductions in the yield and WUE with the RFRH system, but there was a significant increase in the fertilizer use efficiency due to the significant reductions in NO_3_-N and available residual P in the soil. Furthermore, in the dry years (2014 and 2015), R/F2 obtained a similar yield and improved WUE by 10.6% compared with T/F3, where NUE and PUE increased by 13.3% and 12.8%, respectively. Thus, a strategy that reduces the fertilizer rate is recommended for RFRH systems. In addition, R/F2 is a suitable planting strategy for foxtail millet in semiarid areas because it combines a high yield with the sustainable use of resources and the environment. Our results showed that the RFRH system had greater advantages than traditional flat planting in dry years, but an excessively low fertilizer application rate was not conducive to exploiting its potential for increasing the yield and WUE, and an excessively high fertilizer rate led to a low NUE and risk of fertilizer leaching. Therefore, our results provide useful guidance for improving the crop yield and reducing the losses of fertilizer resources in dryland farming throughout the world.

## Conclusion

In the typical semiarid area of the Loess Plateau, wet years are rare and dry years are normal. In the present study, we showed that in dry years, RFRH planting could enhance the WUE and nutrient use efficiency to obtain a higher grain yield compared with T planting under the same fertilizer application rate. In addition, F2 was the most suitable fertilizer application rate for the RFRH system, which obtained a high yield and WUE compared with F3, thereby significantly improving the nutrient use efficiency as well as reducing the NO_3_-N and available P residues in the soil. Thus, the RFRH system combined with F2 (186:96 kg N:P ha^−1^) is recommended as a promising planting strategy for foxtail millet growing in semiarid regions.

## Methods

### Study site description

The field experiments were conducted in three growing seasons from 2013 to 2015 at the Pengyang Dryland Agricultural Research Station, Ningxia Province, China (106°45′N, 35°79′E, 1800 m above sea level), which is characterized as a typical semiarid dryland agriculture region in a hilly and gully area of the Loess Plateau. This region is strongly governed by a semiarid, warm temperate, and continental monsoon climate. The average annual precipitation was determined as 410 mm, approximately 60% of which occurred from July to September, and the average annual free water evaporation was about 1000 mm. The annual temperature average was 8.1 °C and the total duration of sunshine hours was 2518 h year^−1^, with a frost-free period of 155 days. The soil at the experimental site was confirmed as a loess soil with a pH of 8.5, mean bulk density of 1.34 g cm^−3^, average field water-holding capacity (gravimetric) of 22.4%, and a permanent wilting point of 8.3%. Rainfall data were recorded using an automatic weather station (WS-STD1, Delta-T, UK) at the experimental site. The amounts of rainfall during the growth period were 537, 295, and 305 mm in 2013, 2014, and 2015, respectively, and the monthly rainfall distributions are shown in Fig. 5.

### Experimental design and field management

**Figure 6:**
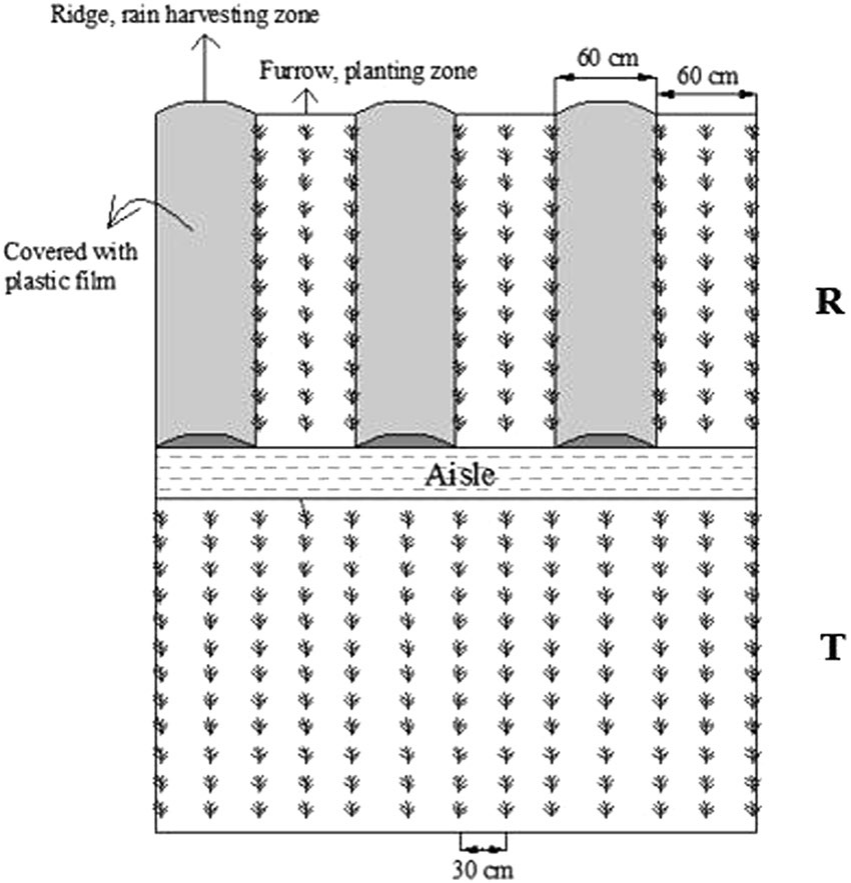
Layout of the field experiments with two planting patterns during the 2013–2015 foxtail millet growing seasons. Note: R = ridge and furrow rainfall harvesting planting; T = traditional flat planting.

Continuous cropping of foxtail millet can cause many problems with land, i.e., weeds, plant diseases, and insect pests, so we selected three different fields for the fertilizer trials in 2013–2015, and the key chemical properties of the 0–40 cm soil layer in each year are listed in Table 5. The experiment was a split plot design with three replicates in each year. The main plots were the two planting patterns (R: RFRH planting, T: traditional flat planting). The subplots received four different fertilizer rates (F0: no fertilizer, F1: N:P at 93:48 kg ha^−1^, F2: N:P at 186:96 kg ha^−1^, F3: N:P at 279:144 kg ha^−1^). The length and width of each plot were 5.0 m × 3.6 m, and they were cultivated by conventional tillage. The RFRH system used ridge and furrow widths of 60 cm, a ridge height of 15 cm, and the ridges were covered with plastic film mulch, where the fertilizer was applied in the furrow area. Schematics of the R and T plots are shown in Fig. 6. The plastic film had a thickness of 0.008 mm (Tianshui Tianbao Plastic Industry Ltd, Gansu, China) and the fertilizer comprised urea (N 46%; China Petroleum Ningxia Petrochemical Production Company) with diammonium phosphate (P_2_O_5_ 46.0%, N 18.0%; Yunnan Three Circles Sinochem Fertilizer Co. Ltd, US-sheng).

**Table 5:** Soil chemical properties in the 0–40 cm soil layer in experimental fields at Pengyang Experimental Station, Ningxia Province, China during 2013–2015.

Year	Soil depth	TN (g kg^−1^)	SOM (g kg^−1^)	AN (mg kg^−1^)	AP (mg kg^−1^)	AK (mg kg^−1^)
2013	0–20 cm	0.86	12.3	52.1	8.8	106
	20–40 cm	0.90	12.7	44.0	3.8	94
2014	0–20 cm	0.98	14.9	63.2	11.8	152
	20–40 cm	0.90	13.4	49.5	4.0	113
2015	0–20 cm	0.85	12.3	33.9	6.5	139
	20–40 cm	0.80	11.8	27.4	3.9	110

Note: TN = total nitrogen; SOM = soil organic matter; AN = available nitrogen; AP = available phosphorus; AN = available potassium.

At 20 days before sowing each year, the entire experimental area was ploughed before marking out the plots. In order to reduce seepage between neighbouring plots, each plot was ridged edgeways and separated by a wide border measuring 60 cm. All of the nitrogen and phosphorus was applied by spreading it evenly over the plot (the whole area of plot for T and the furrows area for R) and ploughing it into the deep soil layer (20 cm) with a spade 10 days before sowing.

Foxtail millet (variety Datong 29) was planted at a rate of 333333 plants ha^−1^. The seeds were sown on April 23, 29, and 24 in 2013, 2014, and 2015, respectively, with an inter-row distance of 30 cm. The foxtail millet was harvested on September 20, 29, and 26 in 2013, 2014, and 2015, respectively. Irrigation was not provided during the experimental years in this study and weeds were controlled manually during each growing season.

### Measurements and data analysis

Soil cores were manually sampled to a depth of 200 cm with increments of 20 cm at the sowing, seedling, jointing, heading, filling, and harvesting stages using a soil ferric auger in 2013–2015. The gravimetric (g g^−1^) SWC of the 0–200 cm profile was measured by drying the soil to a constant weight at 105 °C. If rainfall occurred, we did not sample the soil until the rainwater leakage reached a balance within 2–5 days, which depended mainly on the amount of rainfall. The soil cores were sampled from the middle of a ridge, a furrow, and the side of a furrow in the R plots, where the SWC was calculated as the mean value of the three different positions. In the T plots, the soil cores were taken from the middle of two contiguous rows. SWS was calculated using the following Eq. (1):

1 $${\rm{SWS}}=\sum _{{\rm{i}}}^{{\rm{n}}}{{\rm{c}}}_{{\rm{i}}}\times {{\rm{\rho }}}_{{\rm{i}}}\times {{\rm{h}}}_{{\rm{i}}}/10,$$

where SWS is the amount of soil water storage (mm), c_i_ is the soil gravimetric water content (%), ρ_i_ is the soil bulk density (g cm^−3^), and h_i_ is the soil depth (cm).

Evapotranspiration (ET, mm) for each plot was determined using the soil water balance equation as follows^5^:

2 $${\rm{ET}}={\rm{P}}+{\rm{\Delta }}\mathrm{SWS}-{\rm{D}}-{\rm{R}}+{\rm{G}}+{\rm{I}},$$

where P is the amount of precipitation (mm), ΔSWS is the change in the amount of 0–200 cm soil water storage (mm), D is the downward drainage (mm), R is the surface runoff from each plot (mm), G is the groundwater recharge (mm), I is the irrigation (mm). The experimental field was flat and the groundwater table was at a depth of about 50 m, so no irrigation was required, and I, G, and R in Eq. (2) were zero for all plots. Loess soil has a good water-holding capacity and at the experimental site, the upper 200 cm layer could normally store all of the rainfall; therefore, little drainage occurred below 200 cm, but the SWC at 200 cm exceeded the field water-holding capacity in 2013, and the drainage below 200 cm mainly occurred after strong and continuous rainfall (189 mm rainfall from 81 to 89 DAS). Over 95% of the foxtail millet root system was distributed in the upper 120 cm soil layer^47^, and thus the change in the SWS at the 120–200 cm soil depth during 95 DAS to harvest could be used to estimate the drainage in 2013.

After the harvest, two rows of millet (3 m^2^) were hand harvested from the middle of each plot, where the seed and aboveground biomass yields were determined based on a water content of 12% for the total land area used, including the combined area of the ridges and furrows. WUE was calculated as the foxtail millet yield (kg ha^−1^) divided by the growing season ET (mm).

At the heading and harvesting stages in each year from 2013–2015, soil samples were collected manually to a depth of 200 cm with 20 cm intervals using a 54-mm diameter steel core-sampling tube (T-54, Yangling Machine Equipment Factory, China). For each replicate plot, the sampling positions were in the middle of two contiguous rows for R (furrow area) and T planting, and samples from four points were collected and mixed to obtain a composite profile at the same depth. The soil samples were placed in a plastic box and closed firmly immediately, before being transported and refrigerated (4–6 °C). After the harvest each year, the chemical properties of the soil samples were tested in the same manner. The soil samples obtained at depths of 0–200 cm were used to determine the NO_3_-N content, which was extracted with 2 M KCl solution (20 g fresh soil soaked in 100 ml KCl solution), shaken for 1 h, and analyzed with a FIAstar 5000 Analyzer (FOSS, Sweden). The available phosphorus (Olsen-P) was extracted only from the top soil depths (0–20 and 20–40 cm) using 0.5 M NaHCO_3_ solution adjusted to pH 8.5 and quantified using the Mo-Sb-Vc-method method.

Plant samples were collected during the periods of anthesis and maturity. On each sampling date, six representative foxtail millet plants were collected from each plot under T planting, whereas six plants were selected separately from the side and middle rows under R planting because there was an obvious difference in plant growth in the side and middle rows due to a border effect^48^. The samples obtained at maturity were separated into straw and grain. Tissue samples were subsequently dried to a constant weight in an oven at 65 °C and ground using a plant tissue grinder. After digesting with H_2_SO_4_-H_2_O_2_, the tissue samples were analyzed to determine the N concentration using an automatic Kjeldahl apparatus (FOSS Co., Sweden) and the P concentration was determined using the Mo-Sb-Vc-method method. At anthesis and maturity, the N or P uptake rate by foxtail millet was calculated as the dry matter weight multiplied by the N or P concentration.

NupE (PupE) was calculated as the ratio of N (P) uptake (kg ha^−1^) by the aboveground biomass at maturity relative to the amount of N (P) fertilizer supplied (kg ha^−1^). NUE (PUE) was calculated as the ratio of the grain yield (kg ha^−1^) relative to the total N (P) uptake (kg ha^−1^). NHI (PHI) was calculated as the ratio of grain N uptake relative to the total N (P) uptake^49^.

The amount of N (P) transferred from vegetative organs to grain (Nt, Pt) was calculated using Eq. (3).

3 $$\begin{array}{c}{\rm{Nt}}({\rm{Pt}})={\rm{crop}}\,{\rm{total}}\,{\rm{N}}\,({\rm{P}})\,{\rm{uptake}}\,{\rm{at}}\,{\rm{anthesis}}-{\rm{crop}}\,{\rm{total}}\,{\rm{N}}\,({\rm{P}})\,{\rm{uptake}}\,{\rm{at}}\,{\rm{maturity}}\\ \quad \quad \quad \quad +\,{\rm{grain}}\,{\rm{N}}\,({\rm{P}})\,{\rm{uptake}}\end{array}$$

The contribution rate was calculated as the ratio of Nt (Pt) relative to the grain N (P) uptake.

Net economic income for each treatment was calculated using the following equations:

4 $${\rm{NEI}}=({\rm{GY}}\times {\rm{J}}-{\rm{TC}})\times 0.149$$

5 $${\rm{TC}}=({\rm{fertilizer}}\,{\rm{cost}})+({\rm{plastic}}\,{\rm{film}}\,{\rm{cost}})+({\rm{ridging}}\,{\rm{and}}\,{\rm{mulching}}\,{\rm{cost}}),$$

where NEI is the net economic income (USD ha^−1^), GY is the grain yield (kg ha^−1^), J is the local price of foxtail millet, which was 4 Chinese yuan kg^−1^, 0.149 is the exchange rate (1 Chinese yuan = 0.149 USD, Bank of China, 2017/2/17), and TC is the total cost (Chinese yuan ha^−1^). The local prices of urea and diammonium phosphate were 2 and 3.6 Chinese yuan kg^−1^, respectively, and the total fertilizer cost was calculated based on the fertilizer application rate in different plots. The plastic film cost for the RFRH system was 375 Chinese yuan ha^−1^. Ridging and mulching by machine cost 450 Chinese yuan ha^−1^, and the other costs were the same for all treatments.

The data were tested by analysis of variance using SPSS 22.0, where the data obtained from each sampling event were analyzed separately. Mean values from treatments were compared based on the least significant difference test (LSD 0.05) if the F tests were significant at a probability level of 0.05. All of the figures were prepared using SigmaPlot 10.0.

## References

[1] Wang Q (2015). The optimum ridge–furrow ratio and suitable ridge-covering material in rainwater harvesting for oats production in semiarid regions of China. Field Crops Res..

[2] Chen XL (2012). Rainfall harvesting and mulches combination for corn production in the subhumid areas prone to drought of China. J. Agron. Crop Sci..

[3] Li XY, Gong JD (2002). Effects of different ridge:furrow ratios and supplemental irrigation on crop production in ridge and furrow rainfall harvesting system with mulches. Agric. Water Manage..

[4] Li FM, Guo AH, Wei H (1999). Effects of clear plastic film mulch on yield of spring wheat. Field Crops Res..

[5] Li R (2013). Effects on soil temperature, moisture, and maize yield of cultivation with ridge and furrow mulching in the rainfed area of the Loess Plateau, China. Agric. Water Manage..

[6] Hu Q (2014). Effects of a ridge-furrow micro-field rainwater-harvesting system on potato yield in a semi-arid region. Field Crops Res..

[7] Li X, Su D, Yuan Q (2007). Ridge-furrow planting of alfalfa (Medicago sativa L.) for improved rainwater harvest in rainfed semiarid areas in Northwest China. Soil Tillage Res..

[8] Ding, R. X. *et al*. Optimum width of ridge and furrow for planting foxtail millet in micro-water harvesting systems in arid area of the South Part of Ningxia Province. *Agric. Res. Arid Areas* **25**, 12–16 (2007).

[9] Li CJ (2016). Towards the highly effective use of precipitation by ridge-furrow with plastic film mulching instead of relying on irrigation resources in a dry semi-humid area. Field Crops Res..

[10] Ren XL, Jia ZK, Chen XL (2008). Rainfall concentration for increasing corn production under semiarid climate. Agric. Water Manage..

[11] Deng X-P, Shan L, Zhang H, Turner NC (2006). Improving agricultural water use efficiency in arid and semiarid areas of China. Agricultural Water Management.

[12] Di Paolo E, Rinaldi M (2008). Yield response of corn to irrigation and nitrogen fertilization in a Mediterranean environment. Field Crops Res..

[13] Wang ZQ (2016). Grain yield, water and nitrogen use efficiencies of rice as influenced by irrigation regimes and their interaction with nitrogen rates. Field Crops Res..

[14] Wang X (2015). Water use and soil nitrate nitrogen changes under supplemental irrigation with nitrogen application rate in wheat field. Field Crops Res..

[15] Galloway JN (2008). Transformation of the Nitrogen Cycle: Recent Trends, Questions, and Potential Solutions. Science.

[16] Wang J (2014). Nitrogen and phosphorus leaching losses from intensively managed paddy fields with straw retention. Agric. Water Manage..

[17] Zhong YM (2016). Exploring a suitable nitrogen fertilizer rate to reduce greenhouse gas emissions and ensure rice yields in paddy fields. Sci. Total Environ..

[18] Mcswiney CP, Philip RG (2005). Nonlinear response of N2O flux to incremental fertilizer addition in a continuous maize (Zea mays L.) cropping system. Global Change Biol..

[19] Liu CA (2014). Maize yield and water balance is affected by nitrogen application in a film-mulching ridge–furrow system in a semiarid region of China. Eur. J. Agron..

[20] Cao HX (2007). Mutual physiological genetic mechanism of plant high water use efficiency and nutrition use efficiency. Colloid. Surface. B..

[21] Yang JC (2015). Approaches to achieve high grain yield and high resource use efficiency in rice. Front. Agr. Sci. Eng..

[22] Liu LJ (2014). Combination of site-specific nitrogen management and alternate wetting and drying irrigation increases grain yield and nitrogen and water use efficiency in super rice. Field Crops Res..

[23] Guo SL (2012). Winter wheat grain yield associated with precipitation distribution under long-term nitrogen fertilization in the semiarid Loess Plateau in China. Geoderma.

[24] Wang, C. R., Tian, X. h. & Li, S. X. Effects of cultivation by mulching and rain harvesting on yield and nutrient uptake of winter wheat. *Agric. Res. Arid Areas* **22**, 108–111 (2004).

[25] Li, F. M., Wang, J. & Zhao, S. L. The rainwater harvesting technology approach for dryland agriculture in semi-arid Loess Plateau of China. *Acta Ecol. Sin*. **19**, 117–122 (1999).

[26] Wang, J. P., Han, Q. F., Wang, L. C. & Jia, Z. K. Research on the technique of micro-water harvesting plant in semiarid area of South Ningxia. *Acta Univer. Agric. Boreali-occidentalis* **28**, 16–20 (2000).

[27] Wu Y (2015). Effects of ridge and furrow rainwater harvesting system combined with irrigation on improving water use efficiency of maize (Zea mays L.) in semi-humid area of China. Agric. Water Manage.

[28] Wen XX (2012). Effects of water-collecting and -retaining techniques on photosynthetic rates, yield, and water use efficiency of Millet grown in a semiarid region. J. Integrative Agric..

[29] Li ZZ, Li WD, Li WL (2004). Dry-period irrigation and fertilizer application affect water use and yield of spring wheat in semi-arid regions. Agric. Water Manage..

[30] Liu YL (2015). Agriculture intensifies soil moisture decline in Northern China. Sci. Rep.

[31] Wen ZH (2016). Combined Applications of Nitrogen and Phosphorus Fertilizers with Manure Increase Maize Yield and Nutrient Uptake via Stimulating Root Growth in a Long-Term Experiment. Pedosphere.

[32] Qiu, L. J. *et al*. The effects of different cultivation models and fertilizer application methods on N absorption and translocation of dryland winter wheat. *Plant Nutr. Fert. Sci*. **13**, 355–360 (2007).

[33] Zhao, J. Y. & Yu, Z. W. Effects of nitrogen fertilizer rate on uptake, distribution and utilization of nitrogen in winter wheat under high yielding cultivated condition. *Acta Agron. Sin*. **32**, 484–490 (2006).

[34] Duan, W. X. *et al*. Effects of nitrogen fertilizer application rate on nitrogen absorption, translocation and nitrate nitrogen content in soil of dryland wheat. *Sci. Agric. Sin*. **45**, 3040–3048 (2012).

[35] Mokhtassi-Bidgoli A (2013). Agronomic performance, seed quality and nitrogen uptake of Descurainia sophia in response to different nitrogen rates and water regimes. Ind. Crops Prod..

[36] Sadras VO, Lawson C, Hooper P, Mcdonald GK (2012). Contribution of summer rainfall and nitrogen to the yield and water use efficiency of wheat in Mediterranean-type environments of South Australia. Eur. J. Agron..

[37] Wang Q (2015). Optimum ridge–furrow ratio and suitable ridge-mulching material for Alfalfa production in rainwater harvesting in semi-arid regions of China. Field Crops Res..

[38] Su, W., Qu, Y., Feng, B. L. & Chai, Y. Photosynthesis characteristics and yield of broomcorn millet under film mulching on ridge-furrow for harvesting rainwater model in semi-arid region of Northern Shaanxi. *Trans. Chin. Soc. Agric. Eng*. **30**, 137–145 (2014). 24984496

[39] Ren, X. L., Jia, Z. K. & Chen, X. L. Effect of micro-catchment rainwater harvesting on water and nutrient use efficiency in farmland under different simulated rainfall conditions. *Trans. Chin. Soc. Agric. Eng*. **26**, 75–81 (2010).

[40] Gheysari M (2009). Nitrate leaching in a silage maize field under different irrigation and nitrogen fertilizer rates. Agric. Water Manage..

[41] Robertson GP, Vitousek PM (2009). Nitrogen in agriculture: balancing the cost of an essential resource. Annual Review of Environment and Resources.

[42] Miao YF, Wang ZH, Li SX (2015). Relation of nitrate N accumulation in dryland soil with wheat response to N fertilizer. Field Crops Res..

[43] Han, Q. F. *et al*. Simulated study on soil moisture of field under water micro-collecting farming conditions. *Trans. Chin. Soc. Agric. Eng*. **20**, 78–82 (2004).

[44] Feng, B. *et al*. Effect of nitrogen application level on nitrogen use efficiency in wheat and soil nitrate-N content under bed planting condition. *Acta Agron. sin*. **38**, 1107–1114 (2012).

[45] Li L (2016). Soil Fertility Map for Food Legumes Production Areas in China. Sci. Rep..

[46] Zhang, F. S. *et al*. Nutrent use efficiencies of major cereal crops in China and measures for improvement. *Acta pedol. sin*. **45**, 915–924 (2008).

[47] Liu WH, Sun DZ, Lu B, Bai LX (1996). Research on Laws of Growth and Development of Millet Root System and the Enviornment Effects. Agric. Res. Arid Areas.

[48] Ding, R. X. *et al*. Border effect and physiological characteristic responses of Foxtail Millet to different micro-catchment stripshapes in semi-arid region of south Ningxia. *Sci. Agric. Sin*. 494–501 (2006).

[49] Guo ZJ (2014). Nitrogen use by winter wheat and changes in soil nitrate nitrogen levels with supplemental irrigation based on measurement of moisture content in various soil layers. Field Crops Res..

